# Altruistic Punishment and Impulsivity in Parkinson’s Disease: A Social Neuroscience Perspective

**DOI:** 10.3389/fnbeh.2020.00102

**Published:** 2020-07-21

**Authors:** Rosalba Morese, Sara Palermo

**Affiliations:** ^1^Institute of Public Health, Faculty of Biomedical Sciences, Università della Svizzera italiana, Lugano, Switzerland; ^2^Faculty of Communication, Culture and Society, Università della Svizzera italiana, Lugano, Switzerland; ^3^Center for the Study of Movement Disorders, Department of Neuroscience, University of Turin, Turin, Italy; ^4^European Innovation Partnership on Active and Healthy Ageing, Brussels, Belgium

**Keywords:** impulsive behavior, altruistic punishment, Parkinson’s disease, social cognition, social norms, ingroup and outgroup contexts, default mode network (DMN), functional magnetic resonance imaging (fMRI)

## Abstract

Non-motor symptoms of Parkinson’s disease (PD) are of increasing interest in clinical and psychological research. Disinhibition—the inability to inhibit inappropriate behavior—leads to social and emotional impairments, including impulsive behavior and disregard for social conventions and decision-making behavior. In recent years, the latter has been investigated using economic exchanges during social interactions. Altruistic punishment—to punish someone who violates group norms even if it foresees a personal cost—is one of the most useful and fruitful paradigms; it allows to maintain a cooperation system within social groups. Alterations of this cognitive ability negatively impact the quality of life of the individual and social stability. Social neuroscience has suggested association between impulsive behaviors and altruistic punishment. Neuroimaging research aimed at exploring functional networks and intrinsic functional connectivity went in this direction. To date, little is known about these issues in neurodegenerative diseases such as PD. Dopamine replacement treatment and dopamine-agonists have been associated with impulse-control disorder and impulsive-compulsive behavior able to affect social decision-making. Frontal-executive dysfunction determines an alteration of social functioning through a mechanism of subversion of online action-monitoring, which associates disinhibition with volition. Genetic polymorphisms, alterations of the nigro-striatal substance, and impairment in the medial prefrontal cortex and in the Default mode network (DMN) seem to be able to explain these mechanisms. This theoretical perspective article aims to present these topics in order to encourage an interdisciplinary discussion capable of generating new research and developing rehabilitative intervention to improve social decision-making in PD patients.

## Introduction

This theoretical paper aims to suggest an interdisciplinary vision in Parkinson’s disease (PD) research among different disciplines. Non-motor symptoms in PD are increasingly capturing attention from interdisciplinary research in which psychology, neuroscience, and clinical medicine converge. During the last decades, neuroscientific studies investigated mental processes activated during resting state and social scenarios using economic games (see Sanfey et al., 2006). Theories and experimental paradigms developed by social neurosciences are useful for better understanding impulsivity and decision-making in social situations such as in altruistic punishment, to punish someone who violates group norms even if it foresees a personal cost.

To date, very few studies investigated altruistic punishment in motor neurodegenerative diseases, such as PD. The present theoretical perspective article focuses on common points between impulsivity, metacognitive-executive functions, decision-making processes, and neurobiological factors potentially involved in altruistic punishment in PD patients.

## Altruistic Punishment

Social behaviors arise from “cooperation” which represents a distinctive ability of human beings: that is, the process of individuals and groups acting for their mutual benefit. Cooperation played an important role in the evolution of human social life, allowing the organization in social groups through the creation of social norms (Fehr and Schmidt, 1999; Fehr and Gächter, 2000, 2002; Tomasello, 2009; Boyd et al., 2010; Morese et al., 2018). Joining social groups, respecting their own social norms, has ensured a greater survival in evolutionary history compared to a life in solitude and isolation. Therefore, the transgression has always been sanctioned.

The *altruistic punishment* behavior—to punish someone has carried out an unfair behavior at one’s own cost and with no personal benefit—has been widely studied across several cultures (Fehr and Gächter, 2000, 2002; Gardner and West, 2004; Raihani et al., 2012; Balafoutas et al., 2016; Morese et al., 2016; Rabellino et al., 2016). Djamshidian et al. (2011) underlined how it may have the function of breaking down the amount of unfair behavior within the group. Several authors suggested how altruistic punishment represents the basic nature of cooperation: i.e., cooperation and punishment co-evolve as the one who punishes unfair behavior is considered more reliable, being therefore rewarded for his/her cooperation by the other members of the group (Fehr and Gächter, 2000, 2002; Fehr and Fischbacher, 2004; Gao et al., 2015; Grimalda et al., 2016; Greenwood et al., 2018; Huang et al., 2018). Morese (2018), in line with Henrich et al. (2005), highlights how altruistic punishment is opposed to the classic vision of *homo economicus* guided only by rationality and utilitarian decisions.

Considering the above, altruistic punishment would support emotional processes during social decision-making. Socially driven emotions can be successfully modulated by reappraisal strategies that focus on the reinterpretation of others’ intentions. Indeed, emotion regulation plays a key role in altruistic punishment behavior. According to the theoretical model proposed by Fehr and Gächter (2000, 2002), the altruistic punishment behavior is exercised within the social group and it guarantees the maintenance of cooperation between the members. The experimental paradigm used for the study of altruistic punishment is the Third-Party Punishment (TPP). In this game, a player observes an economic interaction between two other players. One of them can decide to share part of his or her money with the other player, who can only passively accept his or her choice. The player observing the exchange of money can decide whether to punish the behavior if deemed unfair (Morese et al., 2016).

In the last decade, there has been an increasing interest in these issues, while great developments have taken place, thanks to functional neuroimaging techniques (de Quervain et al., 2004; Buckholtz and Marois, 2012; Yang et al., 2019; Zinchenko, 2019).

## Altruistic Punishment and Reward System

The altruist punishment behavior appears to have its neural substrate in the *reward system*. The reward system is a group of neural structures responsible for motivation, associative learning, and positive emotions, especially those involving pleasure as a fundamental component. The thalamus, dorsolateral prefrontal cortex (DLPFC), nucleus accumbens, anterior cingulate cortex (ACC), insula, and caudate nucleus are considered to be part of this neural system (Sanfey et al., 2003; de Quervain et al., 2004; King-Casas et al., 2005; Strobel et al., 2011; Buckholtz and Marois, 2012; Morese et al., 2016; Zinchenko, 2019).

Haber et al. (2006) highlighted the mediation of DLPFC and caudate in punishment responses, being these hubs involved in directing attention toward relevant stimuli, or in understanding communication intentions between individuals. Strobel et al. (2011) discovered that observing unfair behavior evokes the recruitment of the anterior insula—usually activated in the process of disgust—and, therefore, they associated disgust with violation of social norms. Other authors suggested an involvement in brain area usually activated during Theory of Mind (ToM) tasks, such as the medial prefrontal cortex (MPFC) and the temporal-parietal junction (TPJ; Baumgartner et al., 2012; Lo Gerfo et al., 2019). Buckholtz and Marois (2012) proposed the DLPFC, the posterior parietal cortex (PPC), and functional connectivity network [such as the central executive network (CEN)] as involved in decision-making during economic tasks aimed at assigning adequate punishments. More recently, Morese et al. (2016) found the recruitment of the ventral tegmental area (VTA), the MPFC, caudate, and cingulate cortex during tasks eliciting altruistic punishment behavior. VTA plays a central role in the production of dopamine, a neurotransmitter produced in motivation and reward behaviors.

The dopaminergic reward system and VTA are vulnerable in PD, and reward processing abnormalities have been previously identified (Kapogiannis et al., 2011). This might suggest potential altruistic punishment disabilities which must be investigated through a neurocognitive approach (Palermo et al., 2019).

## Parkinson’S Disease, Impulsivity, and Social Decision-Making

PD is a progressive neurodegenerative disorder that affects the central and peripheral nervous systems. Specifically, the depletion of dopaminergic neurons affects the functioning of four fronto-striatal circuits involved in different motor, cognitive, affective, and motivational aspects of behavior (the supplementary motor area, the dorsolateral prefrontal, the orbitofrontal, and the anterior cingulate loops; Palermo et al., 2017a,b, 2018a, 2019; Palermo and Morese, 2018).

Rest tremor, bradykinesia, rigidity, and loss of postural reflexes are generally considered the cardinal signs of PD. Other clinical features include secondary motor symptoms (such as dysarthria, dysphagia, dystonia, festination, freezing, glabellar reflexes, hypomimia, micrographia, shuffling gait, sialorrhoea) and non-motor symptoms (such as autonomic dysfunction, behavioral aberrations, cognitive dysfunctions, sensory abnormalities, and sleep disorders; Jankovic, 2008; Chaudhuri et al., 2011; Palermo et al., 2019). Non-motor symptoms can be more disabling and resistant to treatment than cardinal signs and are key determinants of quality of life in PD (Chaudhuri et al., 2011; Palermo et al., 2019).

As is known, the fundamental therapy for PD is still the pharmacological one, which is implemented with the administration of various active ingredients in addition to levodopa, which remains the most powerful medication, but which presents marked side effects after a few years (Romagnolo et al., 2018; Palermo et al., 2019).

For example, dopamine replacement treatment and dopamine-agonists have been associated with impulse-control disorder, since they can induce changes in those fronto-striatal networks that manage reward and mediate impulse monitoring and control (Ray and Strafella, 2010; Djamshidian et al., 2011). Indeed, tonic stimulation of dopamine receptors damages inhibitory control mechanisms and reward processing while promoting compulsive repetition of behavior (Ray and Strafella, 2010). Impulse-control disorders are associated with appetite disturbance, mood deflection, disinhibition, and irritability (Pontone et al., 2006). Moreover, dysfunction in mental processing speed, shifting between different conceptual sets, and response-inhibition are often encountered, suggesting frontal-executive dysfunction (Palermo et al., 2017a; Palermo and Morese, 2018). All these factors determine an alteration of social functioning through a mechanism of subversion of online action-monitoring, which associates disinhibition with volition. All these factors determine an alteration of social functioning through a mechanism of subversion of online action-monitoring, which associates disinhibition with volition [e.g., pathological gambling; National Research Council (US), 1999; Marazziti et al., 2014].

Disinhibition is habitually considered a synonym for impulsivity (Kocka and Gagnon, 2014). While disinhibition is the background on which euphoria, impulsiveness, and inadequate emotional actions are superimposed (Luria, 1969), impulsivity alters decision-making and motor control in terms of response inhibition (Napier et al., 2015; Palermo and Morese, 2018). A fronto-striatal and cingulo-frontal dysfunction may reflect impairment in metacognitive-executive abilities (such as action-monitoring, response-inhibition, and error awareness; Morese et al., 2018; Palermo et al., 2018b) and promote compulsive repetition of behavior (Palermo et al., 2017a), such as in the case of pathological gambling.

Voon et al. (2011) pointed out an enriched bottom-up ventral-striatal dopamine release to incentive cues, gambling tasks and reward prediction, and possible inhibition of top-down orbito-frontal influences. Thus, dopamine agonist-related ventral-striatal hypo-functionality entails with pounding, medication abuse, hoarding, kleptomania, compulsive shopping, hypersexuality, compulsive eating, and pathological gambling (Voon et al., 2011). Dopaminergic *(dys)*regulation in PD patients with pathological and non-pathological gambling experience has been previously studied using positron emission tomography (Steeves et al., 2009). Neuroimaging findings suggested that patients with pathological gambling exhibit a substantial reduction in the ventral striatum compared to normal controls. The ventral striatum communicates with the limbic and cortical brain structures, being implicated in core regulatory functions such as for motor-like and reward-related behaviors (Steeves et al., 2009). Importantly, Crockett et al. (2010) investigated the relationship among impulsive choice, reward system, and altruistic punishment in economic games, focusing on the role of serotonin. Authors found that a reduction in serotonin levels increased impulsive choice and altruistic punishment behavior. Importantly, the examination of dopamine/glutamate receptors and serotonin transporter gene polymorphisms recognized D3 dopamine receptor p.S9G and GRIN2B c.366C >G as a risk factor for impulse-control disorders in PD (Lee et al., 2009). Genetic polymorphisms may contribute to impulsivity susceptibility (Lee et al., 2009), while modulating social value processing in the striatum, producing context-dependent effects on social decision-making and behavior (Crockett et al., 2013).

## Altruistic Punishment and Parkinson’s Disease

Although dopaminergic dysregulation and its repercussions on social decision-making and behavior are well known, neuroimaging studies evaluating altruistic punishment behavior have not been carried out on PD patients. To date, only the study by Djamshidian et al. (2011) investigates altruistic punishment in PD patients with and without impulsive-compulsive behaviors (ICBs) and healthy participants. Authors adopted an experimental task used based on the research by de Quervain et al. (2004): a trust economic game during which participants must decide whether to punish the fair/unfair behavior of other players. The experimental procedure was simulated through an internet connection so that all participants believed they were “actually” playing with other players. Eight participants played with one trustee per round. In the beginning, each participant received a real sum of £10 which they could decide to give to another player or not. The sum was then quadrupled in each round, and the single player could decide whether to return a portion of the investment to the other participants. At the end, players received £10 more with the option of punishing the other participants but with the clause that the punisher loses £1 for every £2 used to punish. The authors found that PD patients with ICBs punished more than controls on medication, but like controls off medication. These results suggest a role for dopamine in altruistic punishment decisions in PD patients with ICBs (Djamshidian et al., 2011). Indeed, dopaminergic medication can accentuate the desire to enforce social and cooperation rules even if impulsiveness is considered unsuitable for adherence to the group (Djamshidian et al., 2011).

**Figure 1:**
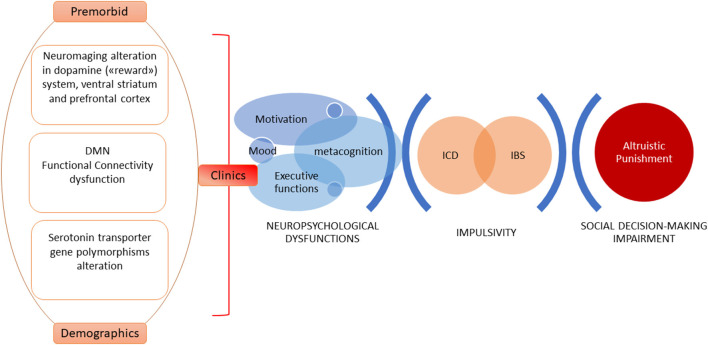
A model of explanation of altruistic punishment impairment in Parkinson’s disease (PD). Complex and widespread cognitive skills such as those related to social cognition require a multidimensional approach. Clinical and demographic predictors, neurobiological and psychobiological factors, and changes in the cognitive-behavioral domain are able to explain impulsive control behaviors characterized by anomalies in reward-driven actions, inhibitory control dysfunctions, and online-monitoring difficulties. These elements—in turn—affect social decision-making, altering the mechanism of altruistic punishment.

Theoretical models explaining altruistic punishment behavior focus on the motivation to punish, trying to discern if it results from a motivational cooperative drive or emotions, such as negative ones (Fehr and Gächter, 2002; Rodrigues et al., 2018). Rodrigues et al. (2018) demonstrated that altruistic punishment can hide negative emotion (anger)—which could be considered as a cover motivational factor. Importantly, PD patients with ICBs can become quite aggressive and have reduced/inexistent self-awareness that their behaviors are unacceptable to others (Djamshidian et al., 2011). Considering the above, new neuroimaging studies on PD patients will have to be designed to explore altruistic punishment neural underpinnings and to discriminate which cover emotions could be able to predict altruistic punishment behavior (see Figure 1).

## Default Mode Network and Altruistic Punishment in Parkinson’s Disease

The default mode network (DMN) is a neural network distributed in different cortical and subcortical regions, which is generally activated during hours of rest and "passive" activities (intrinsic functional connectivity). The cortical and subcortical structures that are part of this resting state network can partly vary from individual to individual, but in general, they are attributable to some main brain areas: the posterior cingulate cortex (PCC), the MPFC, the precuneus, the medial temporal lobe (MTL) and the inferior parietal cortex (IPC), and the ACC (Lucas-Jiménez et al., 2016).

The cognitive skills related to the DMN activation concern are as follows: ability to access memories of one’s life (autobiographical episodic memory), to reflect on one’s own and others’ mental states, to recognize familiar/non-familiar stimuli, and to experience emotions in relation to social situations that concern ourselves or others, to evaluate our own and others’ reactions in some emotional situations. DMN has been found to have a key role also in Third-Party Punishment (TPP), which is explained in a review by Krueger and Hoffman (2016). The authors described the role of three resting state networks elicited during TPP: (1) the salience network (SN), which detects and generates an aversive experience that initiates TPP; (2) the DMN, which integrates the perceived harm and inference of intentions into an assessment of blame; and (3) the CEN, which converts the blame signal into a specific punishment decision.

As explained by Zinchenko and Klucharev (2017), to understand the neural mechanisms of TPP, it is crucial to clarify the neurocomputational mechanism that allows the TPJ (as a part of the DMN) to link norm-violation detection (SN) to specific punishments (CEN).

The association between DMN and the neural basis of social cognition has long been known (Schilbach et al., 2008; Reniers et al., 2012). Hagmann et al. (2008) identified in the TPJ and MFC (as part of the DMN) activations linked to ToM, the ability to attribute mental states—beliefs, intentions, desires, emotions, knowledge—to oneself and to others, and the ability to understand that others have different mental states from their own. In particular, Reniers et al. (2012) reported increased activity in the brain area associated with the DMN during moral decision-making and reduced activation in the DLPFC, when subjecting healthy volunteers to ToM tasks.

PD patients exhibit executive dysfunctions (van Eimeren et al., 2009; Amanzio et al., 2014; Palermo et al., 2017a, 2018a) and ToM disabilities (Palermo et al., 2017a) that are able to explain difficulties in social cognition. Importantly, van Eimeren et al. (2009) supposed a specific DMN malfunctioning during an executive task in PD plausibly linked to dopamine depletion. More recently, Wolters et al. (2019) discussed reduced connectivity in networks related to cognitive impairment and, potentially, affecting social behavior. They found that the DMN was the most prominently involved.

## Conclusions

To understand PD non-motor symptoms, we can allow a multidimensional and personalized approach to patients aimed at enhancing the quality of life (Morese et al., 2018). ICBs—in terms of response-inhibition—has been widely studied applying functional magnetic resonance imaging (fMRI) Go/NoGo paradigm (Braver et al., 2001; Palermo et al., 2018a,b; Gao et al., 2019). This represents a classic experimental design in which a different response frequency is created between responding and not responding to the stimulus (Palermo et al., 2017a,b, 2018b). The conflict is created by the competition between the Go response and the NoGo inhibition response recruiting online monitoring and executive control processes. The fronto-striatal dysfunctions derived by ACC, DPFC, and MPFC hypo-functionality explain executive dysfunction—related to action-monitoring, response-inhibition, and disinhibition responses (Palermo et al., 2017a,b; Palermo and Morese, 2018), which are able to explain also social behavior. Specifically, dysfunctions in these brain areas contribute to impulsivity in PD and metacognitive-executive dysfunctions, potentially involved in altruistic punishment and social decision-making. These evidences have been confirmed not only by fMRI-based paradigm but also by research on resting state networks such as DMN (Schilbach et al., 2008; Reniers et al., 2012; Wolters et al., 2019).

Interdisciplinary perspective could deepen the neurophysiological mechanisms underlying the difficulties in daily life in PD patients. Impulse-control disorders and ICBs can become harmful for PD patients and caregivers, affecting quality of life and social engagement within the contexts of the social groups they belong to Atmaca (2014). We proposed to invest in new frontier bridges between disciplines which will be able to promote new investigation on social cognition in PD. One of the hot topics will certainly be understanding how metacognitive-executive functions and social abilities influence altruistic punishment and TPP in PD.

## Author Contributions

RM conceived the content of the article, wrote the first draft, and reviewed the manuscript. SP wrote the second version of the manuscript, produced infographics, and supervised revision and critiques.

## Conflict of Interest

The authors declare that the research was conducted in the absence of any commercial or financial relationships that could be construed as a potential conflict of interest.
